# Gut microbiota dysbiosis and metabolic shifts in pediatric norovirus infection: a metagenomic study in Northeast China

**DOI:** 10.3389/fcimb.2025.1600470

**Published:** 2025-05-22

**Authors:** Ziju Wang, Xinhong Wei, Lizhen Piao, Xiaofei Zhang, Hong Wang

**Affiliations:** ^1^ Department of Pediatrics, China-Japan Union Hospital of Jilin University, Changchun, Jilin, China; ^2^ Department of Ultrasound, Shandong Provincial Hospital Affiliated to Shandong First Medical University, Jinan, Shandong, China

**Keywords:** norovirus, Northeast China, gut microbial, *Bacteroides uniformis*, metagenomic

## Abstract

**Background:**

Norovirus (NoV) is a leading cause of acute gastroenteritis in pediatric populations worldwide. However, the role of gut microbiota in NoV pathogenesis remains poorly understood.

**Methods:**

We conducted a longitudinal metagenomic analysis of fecal samples from 12 NoV-infected children and 13 age-matched healthy controls in Northeast China. Microbial composition and functional pathways were assessed using high-throughput shotgun sequencing and bioinformatic profiling.

**Results:**

NoV infection was associated with significant gut microbial dysbiosis, including increased alpha diversity and distinct taxonomic shifts. Notably, *Bacteroides uniformis*, *Veillonella* spp., and *Carjivirus communis* were enriched in infected individuals. Functional analysis revealed upregulation of metabolic pathways involved in carbohydrate and lipid processing. These microbial and functional alterations persisted over time and correlated with disease severity.

**Conclusions:**

Our findings reveal novel associations between NoV infection and gut microbiota dysbiosis, particularly the enrichment of *Bacteroides uniformis*, which may influence host-pathogen interactions via metabolic or immune mechanisms. The identified microbial and metabolic signatures offer potential biomarkers for diagnosis and targets for microbiota-based therapeutic strategies in pediatric NoV infection.

## Highlights

- NoV infection induces gut dysbiosis characterized by *Bacteroides uniformis* enrichment and metabolic pathway activation.
- First longitudinal metagenomic profiling of NoV-infected children in Northeast China, linking microbial shifts to functional alterations.
- Identifies potential microbial targets for diagnostics and microbiota-based therapies.

## Introduction

Norovirus (NoV) stands as a principal etiological agent of acute gastroenteritis across global populations. Exhibiting pronounced transmissibility, this pathogen affects all age demographics and demonstrates epidemiological predominance in institutional settings including healthcare facilities, educational institutions, and community environments (Estes et al., 2006; Robilotti et al., 2015). In low- and middle-income countries, NoV accounts for a substantial proportion of diarrheal diseases (Patel et al., 2009; Hall et al., 2013; Ahmed et al., 2014; Flynn et al., 2024). Since the first reported case of NoV infection in China in 1995, sporadic cases and large-scale outbreaks of NoV-induced acute gastroenteritis have been continuously reported in regions such as Beijing, Shanghai, Guangdong, Jiangsu, and Zhejiang (Pan et al., 2016; Xue et al., 2016; Yu et al., 2023). Despite its significant public health impact, progress in developing effective preventive and therapeutic interventions for NoV infection has been impeded by the absence of reliable, scalable *in vitro* platforms and physiologically relevant model systems that can sustain human-derived NoV propagation. Furthermore, the mechanisms underlying NoV infection remain poorly understood, which has limited progress in vaccine and drug development (Zhou et al., 2020).

Colonizing the human gastrointestinal tract at high biomass concentrations, the gut microbiota maintains an expansive genetic repertoire and acts as a pivotal modulator of intestinal mucosal homeostasis (Qin et al., 2010; Adak and Khan, 2019; Zmora et al., 2019). A balanced gut microenvironment is essential for resisting infections caused by enteric pathogens. During childhood, the immature intestinal immune system and relatively unstable colonization resistance make children particularly susceptible to factors that disrupt gut microbial balance, leading to dysbiosis. Studies have shown that most cases of infectious diarrhea in children are closely associated with gut microbiota disturbances (Hempel et al., 2012; Goldenberg et al., 2017). Experimental evidence indicates that gut microbial dysbiosis significantly enhances host susceptibility to rotavirus and other enteric pathogenic microorganisms (Hrncir, 2022). Nevertheless, the mechanistic contributions and functional roles of gut microbiota during norovirus infection remain subjects of ongoing scientific debate. In 2013, Miura et al. first isolated an HBGA-positive Enterobacter cloacae strain from the feces of healthy adults, which could bind to virus-like particles (VLPs) of multiple NoV genotypes (Jones et al., 2014). This finding suggested that certain gut bacteria might facilitate NoV infection by acting as viral co-factors. In contrast, other studies have demonstrated that E. cloacae can inhibit NoV infection in gnotobiotic pigs (Lei et al., 2016b), and certain probiotics have been shown to indirectly suppress NoV infection (Lei et al., 2016a; Lee and Ko, 2017). These contradictory observations underscore the imperative to investigate the dynamic interplay between norovirus infection and enteric microbial communities, with specific emphasis on pediatric populations demonstrating heightened susceptibility to norovirus-induced gastroenteritis.

This investigation centered on pediatric cases of NoV-induced acute gastroenteritis in Northeast China, employing age-matched healthy children as comparative controls. Metagenomic sequencing was utilized to systematically characterize compositional and functional perturbations in the gut microbiota of NoV-infected subjects while establishing baseline microbial profiles for healthy regional populations. The investigation further delineated infection-associated microbial dysbiosis and functional pathway alterations through comparative multi-omics analyses. This work seeks to mechanistically define microbiota-mediated regulatory mechanisms in NoV pathogenesis, yielding innovative perspectives through ecological analysis of host-microbe-virus interactions. The elucidated virus-microbiota crosstalk establishes a mechanistic framework for designing microbiome-based therapeutic strategies to mitigate NoV infection risks in immunocompromised demographics.

## Material and methods

### Sample collection

A total of 25 fecal samples were obtained from children in the Changchun region of Northeast China, including 12 samples from children diagnosed with norovirus (NoV) infection and 13 samples from healthy children as controls. The NoV-positive children were confirmed through clinical diagnosis and laboratory testing, including RT-PCR detection of NoV RNA in fecal samples. Healthy children were selected based on the absence of gastrointestinal symptoms and no history of NoV infection in the past six months. Study cohort stratification systematically accounted for pediatric age, dietary patterns, antibiotic exposure history, and comorbid conditions to ensure group allocation independence from confounding variables. Written informed consent was obtained from the parents or legal guardians of all participants. The study protocol was reviewed and approved by the Ethics Committee of the China-Japan Union Hospital of Jilin University (approval number: 2025012117). Fresh fecal samples were processed in sterile containers, immediately frozen at -80°C, and subsequently transported to the laboratory for further processing and analysis.

### Extraction of microbiome DNA

Genomic DNA was extracted from fecal samples using either the MagPure Stool DNA KF Kit B or the Magnetic Bead Fecal and Soil Genome Extraction Kit (MAGEN, Guangzhou, China), according to the manufacturer’s instructions. In brief, 100–200 mg of fecal sample was placed into a centrifuge tube preloaded with grinding beads, followed by the addition of 1 mL ATL/PVP-10 buffer. Mechanical homogenization was performed using a tissue grinder (Shanghai Jingxin Tech) followed by thermal incubation at 65°C for 20 minutes. Following centrifugation at 14,000 × g for 5 minutes (Eppendorf, Germany), the clarified supernatant was aspirated into fresh reaction vessels. Phase separation was achieved by adding 0.6 mL PCI buffer with 15-second vortex mixing prior to high-speed centrifugation (18,213 × g, 10 minutes). The aqueous phase was combined with magnetic bead binding buffer (600 µL), Proteinase K (20 µL), and RNase A (5 µL) in deep-well plates. Automated nucleic acid isolation was conducted on the KingFisher platform (Thermo Fisher Scientific, USA) using sequential 700 µL aliquots of three distinct wash buffers, culminating in target molecule elution with 100 µL elution buffer. Purified DNA aliquots were cryopreserved at -80°C in 1.5 mL microcentrifuge tubes for downstream analytical processes.

### Metagenomic sequencing

DNA libraries were prepared using the BGI Optimal DNA Library Prep Kit (BGI, Shenzhen, China). Genomic DNA was sheared and size-selected using magnetic bead purification. Fragmented DNA underwent end repair to produce blunt ends, followed by the addition of a 3’-adenine overhang. Sequencing adapters were ligated to the repaired DNA fragments, and the ligated products were PCR-amplified to construct the final library. Rigorous quality control was performed to assess the integrity and uniformity of the final libraries. The double-stranded DNA libraries were denatured to single-stranded DNA and circularized to generate single-stranded circular DNA molecules. Residual linear DNA molecules were eliminated by enzymatic digestion. Each DNB contains approximately 300 tandem copies of the original library molecule. DNBs were loaded onto a patterned nanoarray and sequenced on the DNBSEQ-T7 platform (BGI, Shenzhen, China), generating 150 bp paired-end reads.

### Metagenomic assembly

Raw sequencing reads were quality-filtered and trimmed using SOAPnuke v1.5.2 (Chen et al., 2018), with low-quality reads and adapter sequences removed. Host-derived reads were identified and removed by alignment against the host genome using SOAP2 (Li et al., 2009). High-quality reads were assembled *de novo* using MEGAHIT v1.1.4 (Li et al., 2015), and contigs shorter than 200 bp were excluded from further analysis. Open reading frames (ORFs) were predicted from the assembled contigs using MetaGeneMark (Zhu et al., 2010). Redundant genes were clustered and removed using CD-HIT v4.6.6 (Fu et al., 2012) with identity and alignment coverage thresholds set at 95% and 90%, respectively.

### Data annotation

Gene abundance matrices were generated and quantified using Salmon (v1.5.2) (Patro et al., 2017). Predicted protein sequences were aligned to functional databases, including BacMet, CARD, KEGG, EggNOG, COG, Swiss-Prot, and CAZy, using DIAMOND v0.9.24 (Buchfink et al., 2015) with an E-value threshold of 1e−5. Taxonomic classification was performed using Kraken2 (Wood et al., 2019) based on the lowest common ancestor (LCA) algorithm. For human gut samples, taxonomic annotation was performed against the Unified Human Gastrointestinal Genome (UHGG) v1.0 database (Almeida et al., 2021), which includes more than 200,000 gut microbial genomes. For non-human samples, taxonomic classification was based on the NCBI Nt database (release 202011). Taxonomic and functional abundance profiles were computed using Bracken (https://github.com/jenniferlu717/Bracken) with default settings. Differential abundance analyses of genera, phyla, and KEGG orthologs (KOs) were performed using the Wilcoxon rank-sum test (Matsouaka et al., 2018). Reporter score analysis (Patil and Nielsen, 2005) was applied to identify significantly enriched KEGG pathways, with a significance threshold set at an absolute score of 1.65. Alpha diversity indices, including Shannon, Chao1, and Simpson indices, were calculated at the gene, genus, and KO levels using the vegan R package. Beta diversity was evaluated based on Bray-Curtis dissimilarity (JRBJT, 1957) and Jensen-Shannon divergence (Majtey et al., 2005). All statistical analyses, including Wilcoxon rank-sum and Kruskal-Wallis H tests, were performed in the R environment (v4.0.2).

## Results

### Grouping of norovirus-infected patients and metagenomic quality control in Jilin region

**Figure 1:**
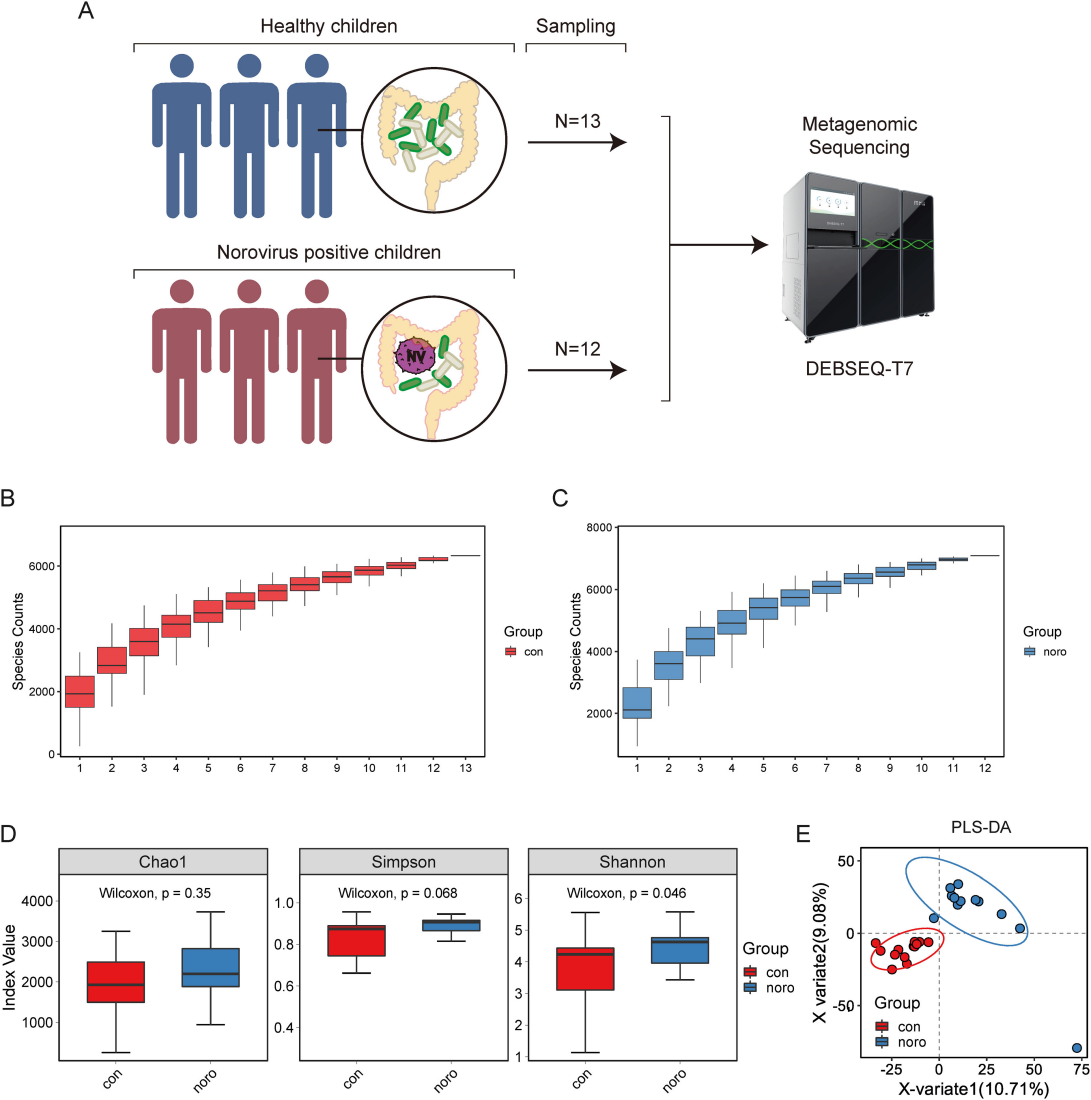
Gut microbiota composition and diversity analysis in healthy versus Norovirus-infected children. **(A)** Workflow of metagenomic sequencing analysis of fecal samples from healthy children (n=13) and Norovirus-infected children (n=12). **(B, C)** Species accumulation curves of gut microbiota in healthy and infected groups. **(D)** Alpha diversity analysis of microbial genes (Shannon index). **(E)** Principal coordinate analysis (PCoA) based on Bray-Curtis dissimilarity between groups (Adonis test, p < 0.05).

This longitudinal study prospectively acquired 25 stool specimens from pediatric subjects in Jilin province during a 24-month surveillance period, stratified into NoV-infected (n=12) and asymptomatic control (n=13) cohorts. These samples were sent to BGI for DNA extraction and metagenomic sequencing using the DEBSEQ-T7 platform (**Figure 1A**). To evaluate and predict the increase in species richness with sample size, species accumulation curves were generated for both healthy and NoV-infected groups (**Figures 1B, C**). The results indicated that the number of species in both groups was approximately 6,000, with a slightly higher species richness observed in the NoV-infected group compared to the healthy group. Quantitative analysis of alpha diversity metrics (Chao1, Shannon, and Simpson indices) enabled systematic comparison of microbial community richness and structural evenness across experimental cohorts. (**Figure 1D**, **Supplementary Table S1**). Consistent with the species accumulation curves, the NoV-infected group exhibited higher alpha diversity than the healthy group, although the difference was not statistically significant. Principal Component Analysis (PLS-DA) was implemented to discern discriminative features in microbiota structural profiles between the two groups (**Figure 1E**). The results revealed that while the two groups were closely positioned on the PLS-DA plot, indicating similar species composition, they were distinctly separated in direction, suggesting notable differences between the groups.

### Comparison of gut microbiota abundance and composition between groups

**Figure 2:**
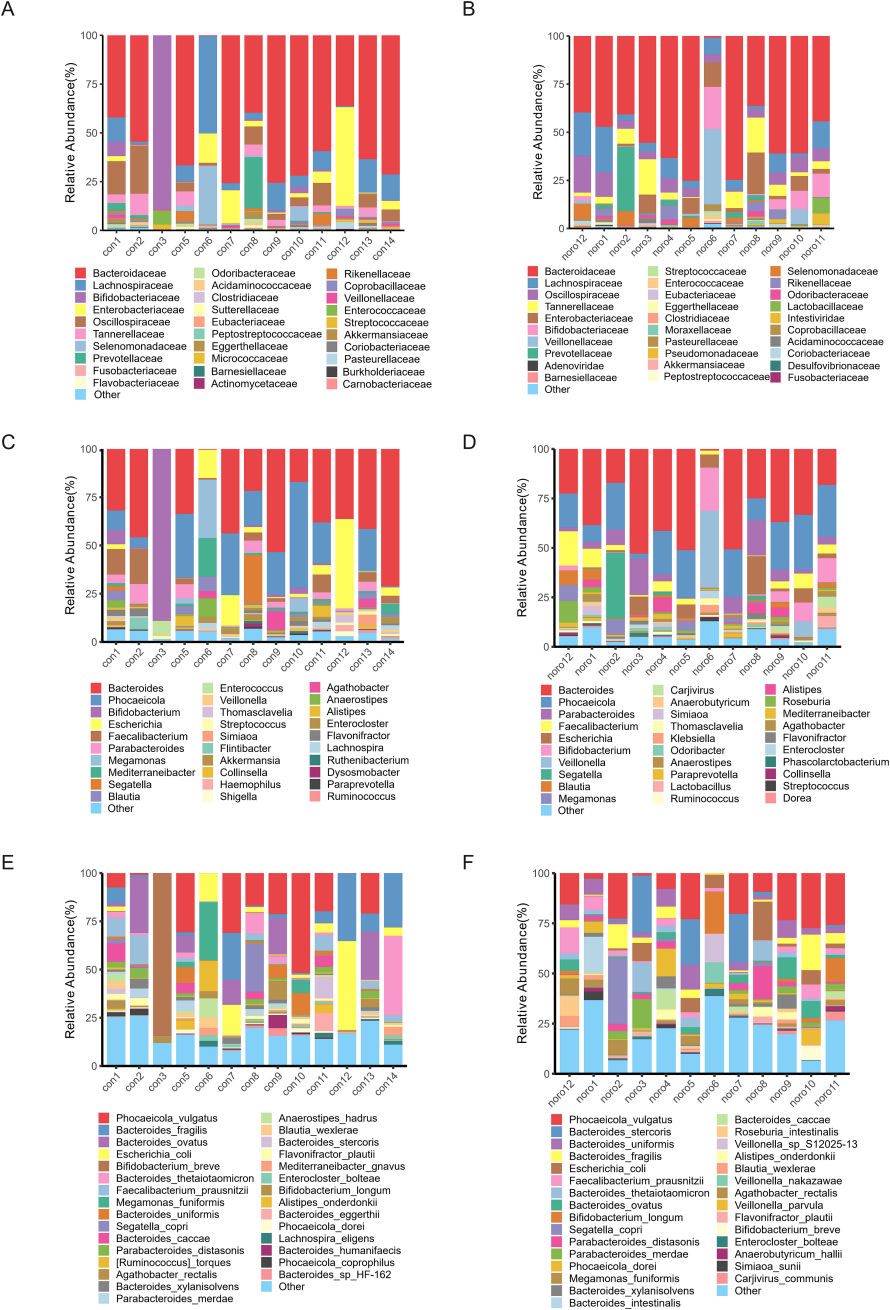
Taxonomic hierarchy of gut microbiota across different classification levels. Relative abundance of gut microbiota at **(A)** Phylum, **(B)** Class, **(C)** Order, **(D)** Family, **(E)** Genus, and **(F)** Species levels in healthy and Norovirus-infected groups.

Species annotation was performed against the NCBI-NR database, and the relative abundance of gut microbiota was calculated at six taxonomic hierarchies (phylum to species) for each individual sample. The top 10 most abundant species were selected to construct stacked bar plots, providing a visual representation of the composition and relative proportions of the major gut microbiota (**Figures 2A–F**). Although individual variation in microbial composition was observed, Bacteroidaceae and Lachnospiraceae were identified as the predominant families in both groups. Notably, sample con3 exhibited a distinct microbial profile dominated by *Bifidobacteriaceae*, which markedly differed from the other samples.

### Comparison of average relative abundance and heatmap visualization between groups

**Figure 3:**
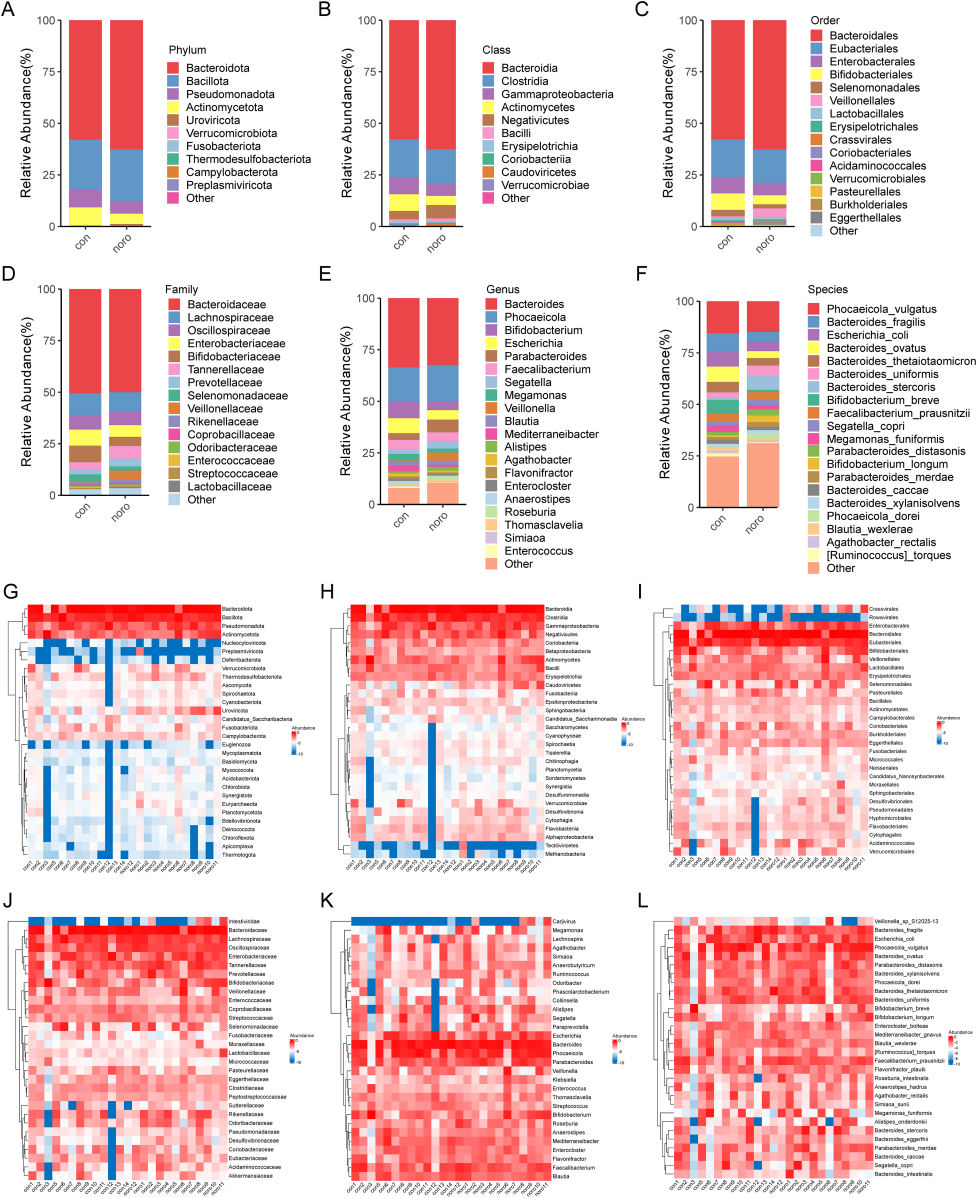
Comparative analysis of gut microbiota taxonomic composition. Mean relative abundance of gut microbiota at **(A)** Phylum, **(B)** Class, **(C)** Order, **(D)** Family, **(E)** Genus, and **(F)** Species levels. Heatmaps **(G-L)** show individual sample variations at respective taxonomic levels. Error bars represent SEM (n=12–13 per group).

To further investigate the average distribution of gut microbiota in healthy and NoV-infected children, the taxonomic abundance at different levels (phylum, class, order, family, genus, and species) was calculated as the group mean (**Figures 3A–F**). Notably, undefined taxa showed upregulation following norovirus infection (**Figures 3E, F**), suggesting potential novel virus-associated microbiota. Cross-phylogenetic analysis revealed that healthy controls exhibited elevated *Bacteroidetes* abundance at phylum, class and order taxonomic ranks compared to NoV-infected subjects. Comparative analysis at the ordinal taxonomic rank revealed significant enrichment of *Eubacterium* populations in healthy controls compared to NoV-infected individuals. Notably, the proportion of unclassified microbial taxa increased at the family, genus, and species levels, especially in the NoV-infected group. Heatmaps were generated to visualize the relative abundance of gut microbiota across different taxonomic levels (**Figures 3G–L**). The results demonstrated that *Bacteroidetes* was the most dominant phylum, while *Tectiliviricetes* exhibited a relatively low abundance. At the family, genus, and species levels, both *Intestiviridae* and *Carjivirus* were identified as low-abundance taxa.

### Lefse analysis and phylogenetic tree of differential microbiota

**Figure 4:**
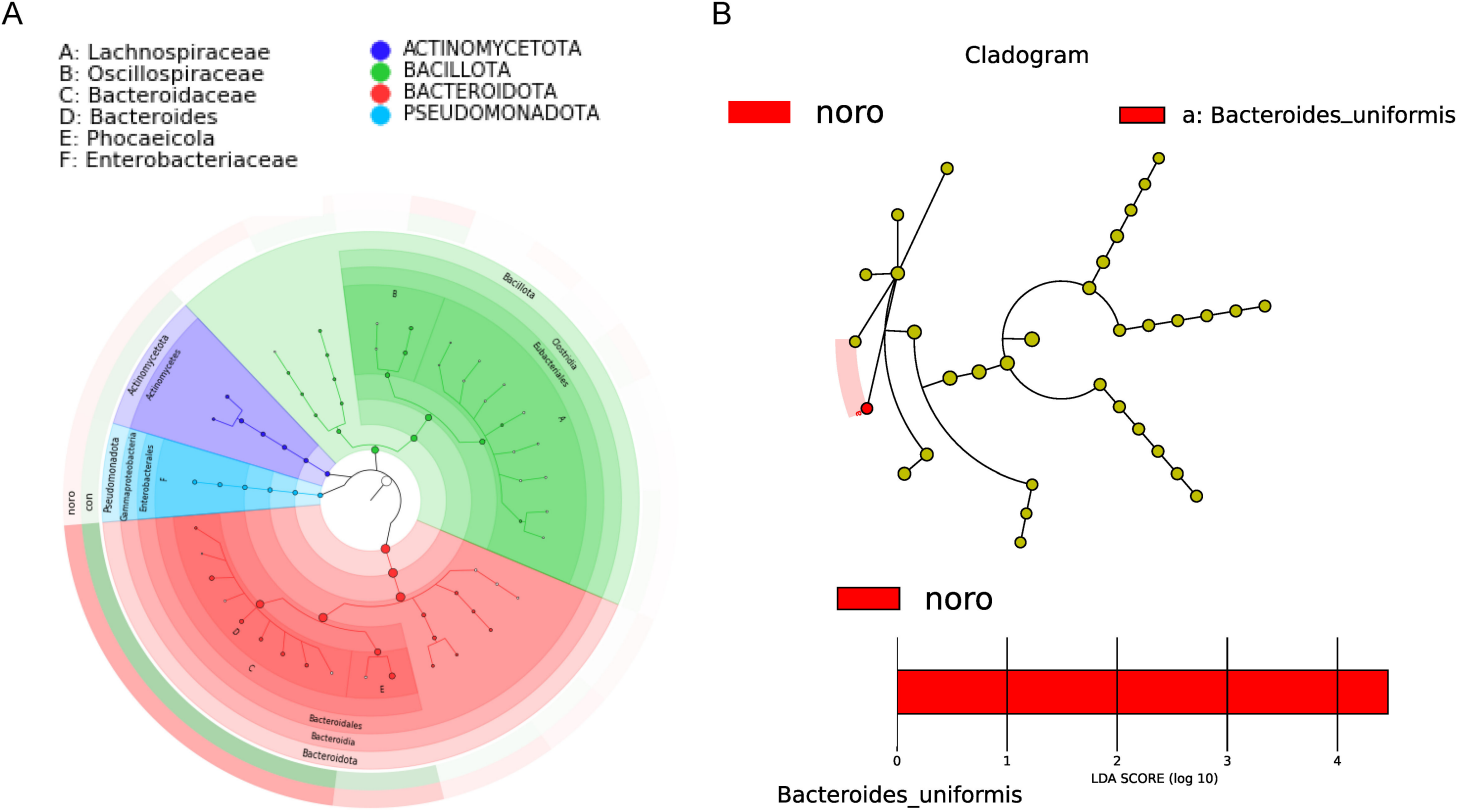
Phylogenetic and biomarker analysis of gut microbiota. **(A)** GraPhlAn phylogenetic tree of dominant taxa in healthy and infected groups. **(B)** LEfSe analysis identifying differentially abundant taxa (LDA score >3.0, p < 0.05 by Kruskal-Wallis test).

A GraPhlAn phylogenetic tree was constructed to illustrate the evolutionary relationships of microbiota between the healthy and NoV-infected groups (**Figure 4A**, **Supplementary Table S2**). The tree revealed that *Actinomycetota, Bacillota, Bacteroidota*, and *Pseudomonadota* were the core microbiota with clear evolutionary relationships. LEfSe was employed to detect differentially abundant species across groups (LDA >3), revealing *Bacteroides uniformis* as the most discriminative taxon (LDA >4) in NoV-infected individuals (**Figure 4B**).This suggests that changes in *Bacteroides uniformis* may be closely associated with NoV infection.

### Relative abundance and confidence interval analysis of differential microbiota

**Figure 5:**
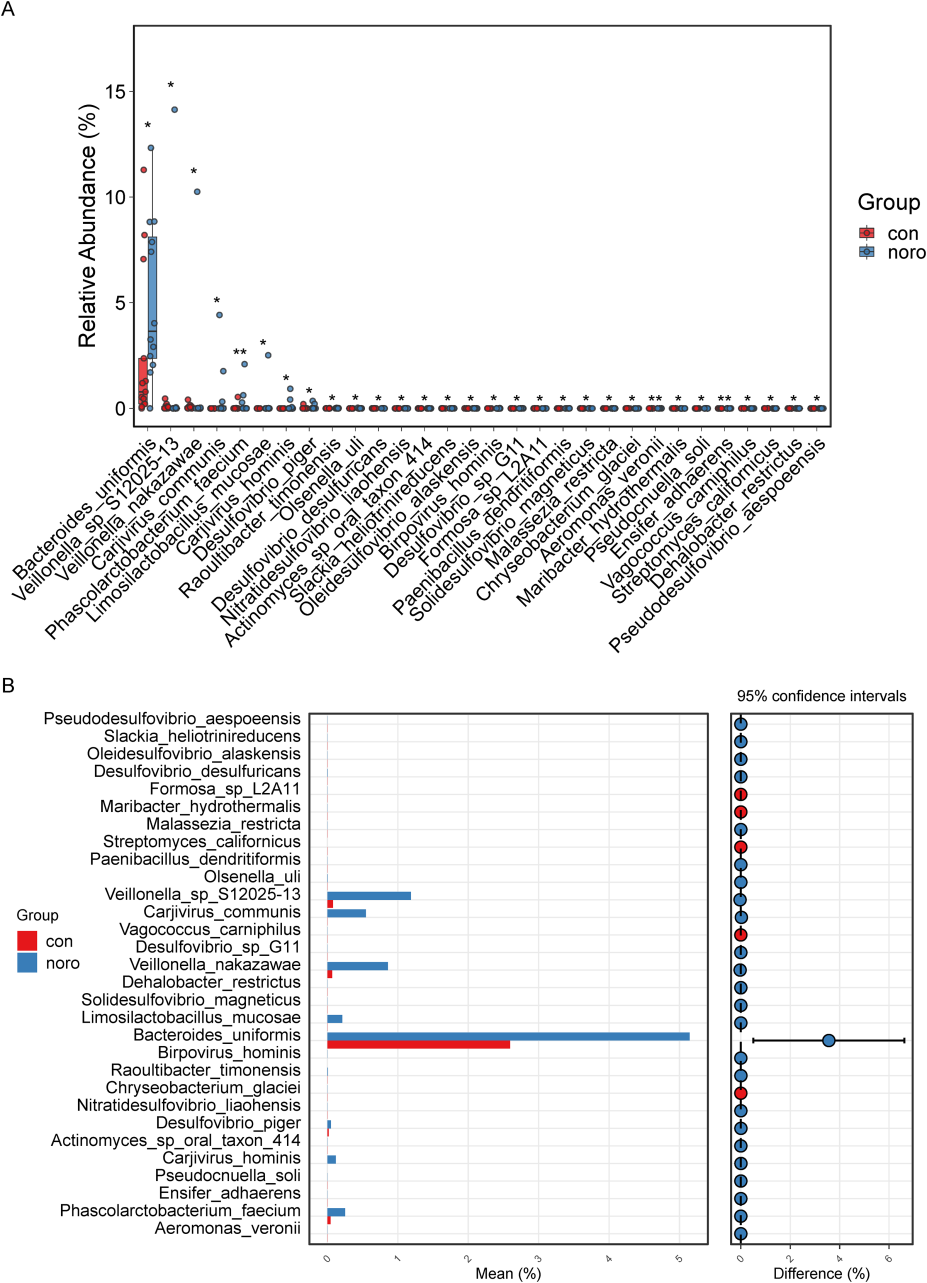
Differential microbial taxa between groups. **(A)** Mean expression levels of significantly altered taxa (Wilcoxon rank-sum test, *p < 0.05). **(B)** Confidence interval analysis of differential taxa (95% CI, Benjamini-Hochberg FDR correction).

Taxa with significant inter-group abundance variations were identified and quantified, revealing a >3-fold increase in *Bacteroides uniformis* in NoV-infected individuals (**Figure 5A**, **Supplementary Table S2**). Other taxa, including *Veillonella* sp. *S12025-13, Carjivirus communis*, and *Veillonella nakazawae*, also exhibited significant differences, suggesting their potential roles in NoV infection. Additionally, the mean values and confidence intervals of differential microbiota were calculated (**Figure 5B**). The results confirmed that *Bacteroides uniformis* had the highest confidence interval and degree of difference, while *Veillonella* sp. *S12025-13, Carjivirus communis, Veillonella nakazawae*, and *Limosilactobacillus mucosae* also showed significant upregulation in the NoV-infected group.

### Functional and metabolic pathway analysis of gut microbiota

**Figure 6:**
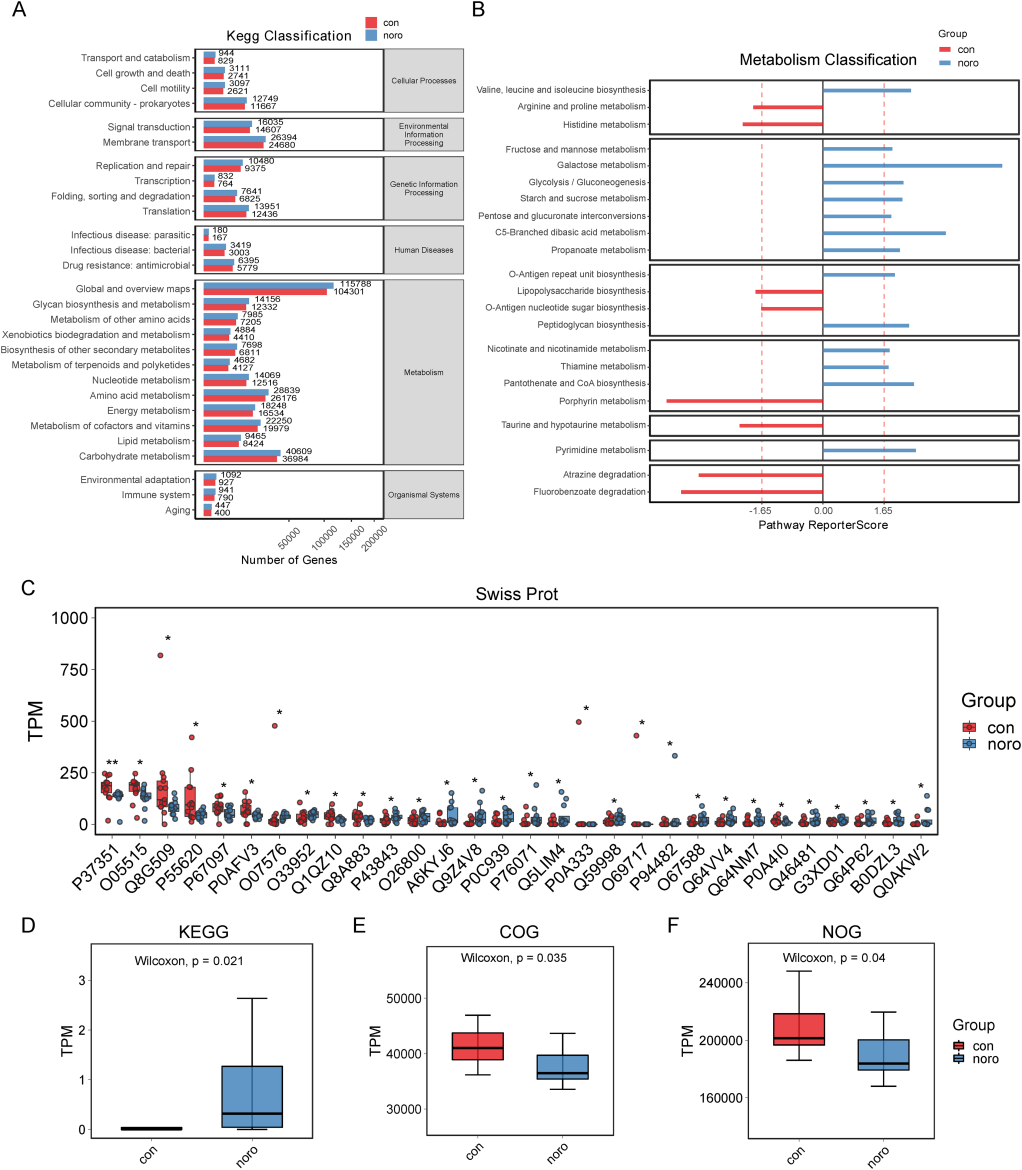
Functional profiling of gut microbiota. **(A)** KEGG pathway enrichment analysis. **(B)** Metabolic function clustering (DAVID analysis, FDR <0.1). **(C)** Swiss-Prot protein domain enrichment. **(D-F)** Enrichment scores for KEGG, COG, and NOG databases (*p < 0.05 vs. healthy group).

KEGG pathway analysis demonstrated a broader functional gene repertoire in the NoV-infected cohort, with significant enrichment in carbohydrate, lipid, and cofactor/vitamin metabolism compared to healthy controls (**Figure 6A**, **Supplementary Table S3**). This metabolic reprogramming suggests a potential adaptation to support viral replication or host immune evasion. Detailed metabolic profiling (**Figure 6B**, **Supplementary Table S4**) revealed upregulated pathways in the NoV-infected group, including galactose utilization, C5-branched dibasic acid processing, and branched-chain amino acid (valine, leucine, isoleucine) biosynthesis. In contrast, the healthy group exhibited enrichment in pathways such as porphyrin metabolism, fluorobenzoate degradation, and atrazine degradation. Swiss-Prot pathway analysis (**Figure 6C**) revealed downregulation of bacterial proteins such as P7351, O05515, Q8G509, and P55620 in the NoV-infected group, while proteins like A6KYJ6, Q9Z4V8, P0C939, P76071, and Q5LIM4 were upregulated. Finally, quantitative scoring of KEGG, COG, and NOG enrichment (**Figures 6D–F**) showed that the NoV-infected group had significantly higher KEGG TPM values but lower COG and NOG TPM values compared to the healthy group, indicating that NoV infection primarily affects KEGG pathways while reducing COG and NOG pathway enrichment.

## Discussion

This investigation provides revelations regarding enteric microbiome architecture and metabolic flux patterns in pediatric norovirus infections within northeastern Chinese populations., compared to healthy controls, revealing key taxa and pathways associated with disease pathogenesis. The gut microbiota in both groups was predominantly composed of *Bacteroidaceae* and *Lachnospiraceae*, which aligns with previous studies demonstrating the dominance of these families in the human gut microbiota (Zhernakova et al., 2016). However, our analysis revealed notable differences in specific bacterial taxa and metabolic pathways between NoV-infected and healthy children, suggesting a potential link between gut microbiota dysbiosis and NoV infection. These findings are consistent with emerging evidence that viral infections, including NoV, can significantly alter the gut microbial ecosystem, potentially influencing disease outcomes (Khalil et al., 2024). For instance, studies have shown that NoV infection can lead to shifts in the abundance of *Bacteroides* and *Veillonella* species, such alterations could potentially modulate immune responses or enhance viral propagation (Zhou et al., 2021; Pither et al., 2024). New evidence demonstrates *Rhodococcus* exhibits antiviral activity against human norovirus through clinical specimen interactions and enteroid replication suppression (Santiso-Bellon et al., 2025). Our analysis further indicates geographic/ethnic population-specific variations in norovirus-linked microbial consortia.

One of the most notable findings was the significant difference in *Bacteroides uniformis* abundance between the two groups. *Bacteroides* species are crucial for maintaining gut homeostasis, and their dysregulation has been linked to various gastrointestinal infections (Zafar and Saier, 2021). The altered levels of *Bacteroides uniformis* in NoV-infected children may indicate a disruption in gut microbial equilibrium, potentially exacerbating inflammation or compromising mucosal barrier integrity. It is crucial to acknowledge the methodological constraint regarding *Bacteroides uniformis*-NoV infection association, as causal inference remains unsubstantiated by direct experimental evidence. Furthermore, significant differences in the abundance of *Veillonella* sp. *S12025-13, Carjivirus communis*, and *Veillonella nakazawae* emphasize the influence of NoV infection on gut microbiota composition. These results align with prior studies showing that NoV infection can cause shifts in specific bacterial taxa, such as *Enterobacter* and *Veillonella*, which may affect viral binding or host immune responses (Yen et al., 2014). For instance, Miura et al. (2013) reported that *Enterobacter cloacae* could bind to NoV virus-like particles (VLPs), indicating a potential role for certain bacteria in promoting NoV infection (Miura et al., 2013). Conversely, other studies have shown that *Veillonella* species may modulate immune responses or compete with pathogens for resources, potentially exerting protective effects (Zhou et al., 2021). Interestingly, post-norovirus infection analysis revealed enrichment of uncharacterized microbial taxa, indicating putative virus-specific microbiota candidates that warrant culture-based isolation and taxonomic definition. The contrasting roles of these bacteria in NoV infection underscore the complexity of host-microbe-virus interactions and warrant further investigation.

Functional metagenomic characterization demonstrated marked activation of nutrient processing pathways, particularly in carbohydrate utilization, lipid biosynthesis, and vitamin-associated cofactor cycling among NoV-infected subjects. These results suggest that NoV infection may induce metabolic reprogramming of the gut microbiota, either as an adaptive response to the altered intestinal environment or to promote viral replication. The upregulation of these metabolic pathways may reflect increased energy and nutrient demands in the infected host, likely driven by immune responses to the viral pathogen. These observations align with established virome-microbiome crosstalk mechanisms demonstrating that viral infections, including NoV, can significantly alter the metabolic potential of the gut microbiota, often promoting pathways that facilitate pathogen survival or evasion of host immunity (Belliot et al., 2014; Cates et al., 2020). For instance, NoV infection in mice has been linked to increased expression of genes associated with carbohydrate and lipid metabolism, suggesting that the virus may exploit host metabolic processes to enhance its replication (Hassan and Baldridge, 2019). Similarly, our results corroborate previous findings that NoV infection can modulate the gut microbiome functional remodeling, potentially contributing to increased disease severity or prolonged infection (Nelson et al., 2012; Axelrad et al., 2023).

The distinct alterations in specific bacterial taxa and metabolic pathways identified in this study provide a foundation for future research into the mechanisms underlying gut microbiota dysbiosis during viral infections. Further investigations are required to determine whether these microbial alterations act as causative factors in NoV pathogenesis or represent secondary consequences of viral infection. Additionally, microbiota-targeted interventions, including probiotics and dietary modifications, show considerable potential as therapeutic strategies to alleviate NoV-associated symptoms. Deciphering the pathogen-microbiome interface dynamics through multi-omics approaches provides critical insights for devising targeted microbial therapeutics to mitigate clinical burdens of pediatric norovirus infections in endemic regions. By comparing our findings with existing literature, we observed both concordances and discrepancies, highlighting the need for further studies to clarify the roles of specific bacterial taxa and metabolic pathways in NoV infection. This study adds to the growing body of evidence that implicates the gut microbiota as a critical modulator of host-virus interactions, offering novel opportunities for microbiota-based therapeutic interventions.

In summary, this study highlights the substantial alterations in gut microbiota composition and functional potential associated with NoV infection in children. The identified shifts in specific bacterial taxa and metabolic pathways lay a foundation for further studies into the mechanisms underlying gut microbiota dysbiosis during viral infections. Future research should clarify whether these microbial shifts constitute initiating factors of NoV pathogenesis or represent adaptive responses to host-pathogen interactions. Moreover, systematic assessment of microbiota-directed therapeutic approaches, including probiotic supplementation and dietary modulation, in reducing NoV-associated morbidity presents a promising therapeutic avenue. Deciphering the dynamic crosstalk mechanisms between norovirus and gut microbial ecosystems holds promise for pioneering prophylactic approaches and precision interventions against this globally prevalent childhood enteric pathogen. Comparison of our findings with previous studies revealed both consistencies and discrepancies, underscoring the need for further investigation into the specific roles of bacterial taxa and metabolic pathways in NoV infection. These findings contribute to accumulating evidence delineating the gut microbiome’s central regulatory function in antiviral defense mechanisms, while providing novel therapeutic frameworks for microbial ecosystem engineering in infectious disease management.

## Data Availability

The datasets presented in this study can be found in online repositories. The names of the repository/repositories and accession number(s) has been deposited in the National Center for Biotechnology Information Sequence Read Archive (BioRrgisct: PRINA1209515) underopen-access provisions.
